# Genome-wide linkage and association analysis of rheumatoid arthritis in a Canadian population

**DOI:** 10.1186/1753-6561-1-s1-s19

**Published:** 2007-12-18

**Authors:** Zhi Wei, Mingyao Li

**Affiliations:** 1 Genomics and Computational Biology Graduate Group, University of Pennsylvania School of Medicine, 1401 Blockley Hall, 423 Guardian Drive, Philadelphia, Pennsylvania 19104, USA; 2 Department of Biostatistics and Epidemiology, University of Pennsylvania School of Medicine, 629 Blockley Hall, 423 Guardian Drive, Philadelphia, Pennsylvania 19104, USA

## Abstract

Rheumatoid arthritis (RA) is an autoimmune disease with a moderately strong genetic component. Previous linkage and candidate gene studies have identified several regions that predispose to RA, including the *HLA-DRB1 *and *PTPN22*. We conducted genome-wide linkage analysis with 128 affected individuals from 60 families in a Canadian cohort that were genotyped using the Illumina linkage panel and genome-wide association analysis with 158 affected individuals from the same cohort that were genotyped using the Affymetrix 100 K platform. Multipoint nonparametric linkage scan revealed three linkage peaks with LOD scores greater than 1.5. We also identified 13 significantly associated SNPs at the genome-wide level of 0.05 after Bonferroni adjustment for multiple testing. Several of the significantly associated SNPs are located close to previously identified linkage regions, but not in the linkage peaks identified in the same cohort. We could not replicate association with *HLA-DRB1 *and *PTPN22*. Our results indicate that high coverage and sufficient sample size are crucial for the success of genome-wide association studies.

## Background

Rheumatoid arthritis (RA) is a complex autoimmune genetic disorder in which the immune system attacks normal tissues as if they were invading pathogens. Twin and family studies have suggested that the heritability of RA is ~60%. A well established RA susceptibility locus is the HLA region located on chromosome 6p, which is estimated to account for one-third of the genetic component of RA etiology. Apart from the HLA region, a number of other chromosomal regions have been replicated among various genome-wide linkage scans in which the leading regions include chromosome 1p13, 1q41–43, 6q16, 16p, and 18q [1].

Linkage analysis has low power to detect genetic variants that confer modest disease risks. For complex diseases such as RA, tests of genetic association with the disease may be more effective. Genetic association analyses have led to the identification of *PTPN22 *[2], a gene that has been replicated in many subsequent studies. Additional susceptibility loci for RA that have been implicated by association analyses include *PADI4*, *SLC22A4*, *RUNX1*, and *CTLA4*.

In this investigation, we performed genome-wide linkage and association analyses of the Canadian Rheumatoid Arthritis Genetic Study (CRAGS) data made available to Genetic Analysis Workshop 15 participants. We seek to identify genetic variants that predispose to RA and to characterize their genetic contributions.

## Methods

### Data sets and initial data quality checking

The CRAGS provided two data sets. The first data set includes 60 families (128 affected individuals) that were genotyped using the Illumina linkage panel on 5429 SNPs across 22 autosomal chromosomes. The second data set includes 158 affected individuals (78 affected sib pairs (ASPs) and one affected avuncular pair) that were genotyped using the Affymetrix 100 K platform on 113,237 SNPs across 22 autosomal chromosomes. Among the 113,237 SNPs, a total of 87,181 SNPs had >85% genotypes completed, and exhibited a minor allele frequency (MAF) of >0.05. The 87,181 SNPs that passed the initial quality control had an average MAF of 0.247 and genotyping success rate of 96.8%.

### Test of Hardy-Weinberg equilibrium in the presence of disease association

Assessing Hardy-Weinberg equilibrium (HWE) is often an important step for checking the quality of genotype data. The standard test of HWE assumes that the genotypes are randomly sampled from the general population. However, in the CRAGS, all individuals are affected. As a result, when a marker is associated with the disease, the corresponding genotypes may no longer be a random sample. Assessing departure from HWE in the presence of disease association is particularly important for genome-wide association studies in which the disease variants are either directly genotyped or are in linkage disequilibrium (LD) with the genotyped markers. Analysis using the standard HWE test might result in many rejections, and perhaps, some of the rejected markers are in LD with the disease variants. Here we develop a likelihood framework that allows the assessment of departure from HWE while taking into account potential association with the disease.

Assume a homogeneous sample of ASPs is collected and genotyped at a diallelic marker with two alleles *A *and *a *(with frequencies *p *and *q*, respectively). Let *g *∈ {0, 1, 2} represent the number of allele *A*, and *P*_*g *_be the corresponding genotype frequency. Under HWE, the genotype frequencies in the general population are *P*_0 _= *q*^2^, *P*_1 _= 2*pq*, and *P*_2 _= *p*^2^, respectively. For an ASP with genotypes *g*_1 _and *g*_2_, the retrospective likelihood is $\mathrm{Pr}(g_{1},g_{2}|ASP)=r_{g_{1}}r_{g_{2}}\mathrm{Pr}(g_{1},g_{2})/\sum_{\left(g_{1,}g_{2}\right)}r_{g_{1}}r_{g_{2}}\mathrm{Pr}(g_{1},g_{2})$, where *r*_*g *_is the genotype relative risk of genotype *g *compared with genotype 0. When HWE is assumed, the parameters to be estimated are {*r*_1_, *r*_2_, *p*}; when departure from HWE is allowed, the parameters to be estimated are {*r*_1_, *r*_2_, *P*_1_, *P*_2_}. Table 1 lists the joint genotype probability for a sib pair under the null and the alternative models, respectively. For a sample of ASPs, the overall likelihood of the data, *L*, is simply the product of the likelihood for each ASP. We can test for residual departure from HWE using a likelihood ratio statistic *T *= 2[ln($\hat{L}$_1_) - ln($\hat{L}$_0_)], where $\hat{L}$_1 _and $\hat{L}$_0 _are the likelihoods maximized under the alternative and the null models, respectively. Under the null hypothesis of HWE, *T *is approximately distributed as a χ^2 ^distribution with one degree of freedom. This test assesses departure from HWE after adjusting for possible association with the disease, therefore minimizing the chance that an important marker is flagged as problematic.

**Table 1:** Joint genotype probability for a sib pair (genotypes are unordered)

	Pr(*g*_1_, *g*_2_)	
	
(*g*_1_, *g*_2_)	Assume HWE	Allow departure from HWE
(0, 0)	$q^{4}+pq^{3}+\frac{1}{4}p^{2}q^{2}$	$P_{0}^{2}+\frac{1}{2}P_{0}P_{1}+\frac{1}{16}P_{1}^{2}$
(0, 1)	*pq*^2^(1 + *q*)	$P_{0}P_{1}+\frac{1}{4}P_{1}^{2}$
(0, 2)	$\frac{1}{2}p^{2}q^{2}$	$\frac{1}{8}P_{1}^{2}$
(1, 1)	*pq*(1 + *pq*)	$\frac{1}{2}P_{0}P_{1}+\frac{1}{4}P_{1}^{2}+\frac{1}{2}P_{1}P_{2}+2P_{0}P_{2}$
(1, 2)	*p*^2^*q*(1 + *p*)	$\frac{1}{4}P_{1}^{2}+P_{1}P_{2}$
(2, 2)	$\frac{1}{4}p^{2}q^{2}+p^{3}q+p^{4}$	$\frac{1}{16}P_{1}^{2}+\frac{1}{2}P_{1}P_{2}+P_{2}^{2}$

### Linkage and association analysis

We performed genome-wide, nonparametric multipoint linkage analysis using the S_PAIR_ statistic [3] as implemented in MERLIN [4] on the 60 families that were genotyped using the Illumina linkage panel. The S_PAIR_ statistic combines information from pairs of affected individuals and can detect regions of excess IBD sharing.

We performed single-marker association analysis using LAMP [5,6], which uses a maximum-likelihood model to extract information on genetic association from samples of unrelated individuals, sibships, and larger pedigrees. Briefly, the program estimates the disease-SNP haplotype frequencies and three penetrances using all available data by maximizing the likelihood of the marker data conditional on the disease phenotypes. A likelihood ratio test with approximately two degrees of freedom is constructed by comparing the likelihood maximized under the alternative model, which allows for LD between the disease and SNP loci, with the likelihood maximized under the null model that assumes linkage equilibrium. We assumed a fixed disease prevalence of 0.8%. Different disease prevalence changed parameter estimates slightly, but did not appear to affect the overall ranking of SNPs.

## Results

**Figure 1:**
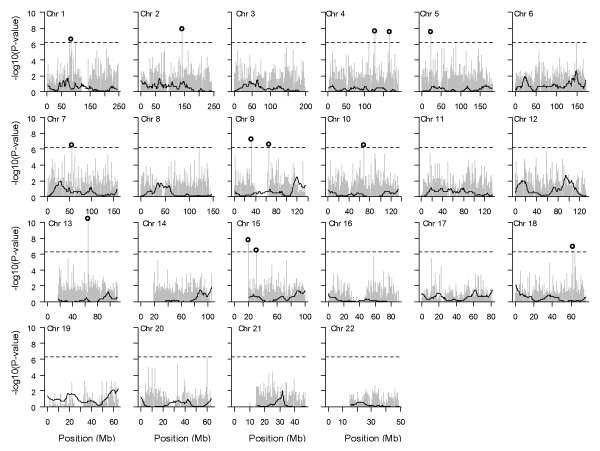
**Genome-wide linkage and association analysis**. The solid curve is the -log10(*p*-value) of the multipoint LOD score from MERLIN. The gray line is the -log10(*p*-value) of the likelihood ratio test of association from LAMP. SNPs that are significantly associated with RA after Bonferroni adjustment are circled.

Our multipoint nonparametric linkage analysis revealed three linkage signals at a LOD score threshold of 1.5, corresponding to a -log10(*p*-value) > 2.37 (Figure 1). These linkage peaks are on chromosome 12 (LOD = 1.89 at 123 cM, asymptotic *p*-value = 0.002), chromosome 6 (LOD = 1.83 at 161.7 cM, asymptotic *p*-value = 0.002), and chromosome 9 (LOD = 1.69 at 141 cM, asymptotic *p*-value = 0.003). We did not observe evidence of linkage in the HLA region, despite the fact that approximately one-third of the total genetic contribution in RA is attributed to genes in the HLA region. Because the Affymetrix 100 K platform includes a denser set of SNPs in the HLA region and more ASPs in the CRAGS were genotyped, we also conducted nonparametric linkage analysis on chromosome 6 with the Affymetrix SNPs. The analysis was conducted using MERLIN [4], in which LD between SNPs was modeled by considering haplotypes within clusters of tightly linked markers. We obtained results similar to those from the Illumina SNPs, suggesting that the lack of linkage evidence is probably due to the limited sample size of the CRAGS.

Among the 87,181 SNPs that were genotyped by the Affymetrix 100 K platform and passed initial data quality checking, 145 of them had a *p*-value < 0.001 using our test of HWE. These SNPs were excluded from subsequent association analysis because LAMP assumes HWE at the tested SNP in the general population. For the remaining 87,036 SNPs, we did single-marker association analysis using LAMP (Figure 1). We corrected for multiple testing using the Bonferroni criterion and controlled the family-wise error rate at α_genome _= 0.05. We identified 13 significantly associated SNPs at the genome-wide level, but none of them fell in linkage peaks identified using the 60 families (Table 2).

**Table 2:** Significantly associated SNPs after Bonferroni correction with α_genome _= 0.05 using LAMP

Chr	SNP	Position (bp)	MAF	LRT	*p*-Value
1	rs12564931	83,251,950	0.3028	30.49	2.39 × 10^-7^
2	SNP_A-1732798	142,778,205	0.0784	36.24	1.35 × 10^-8^
4	rs4834009	126,300,977	0.1523	35.00	2.51 × 10^-8^
4	rs10517834	166,748,108	0.3014	34.77	2.82 × 10^-8^
5	rs10520893	23,724,883	0.0839	34.49	3.24 × 10^-8^
7	rs6593179	54,518,391	0.2803	29.98	3.09 × 10^-7^
9	rs680471	32,506,949	0.2953	33.43	5.05 × 10^-8^
9	rs4111290	66,457,420	0.2821	30.16	2.82 × 10^-7^
10	rs10509272	67,769,079	0.2739	29.75	3.47 × 10^-7^
13	rs10492477	65,139,738	0.1029	48.26	3.31 × 10^-11^
15	rs2090622	19,204,681	0.3038	35.69	1.78 × 10^-8^
15	rs10519774	31,024,477	0.2566	29.79	3.40 × 10^-7^
18	rs1115947	62,064,985	0.2853	32.00	1.13 × 10^-7^

The most strongly associated SNP is rs10492477, located at 13q21. This SNP maps to the *PCDH9 *gene, which belongs to the protocadherin gene family, a subfamily of the cadherin superfamily. *PCDH9 *is predominantly expressed in brain, but is also expressed in hairy cell leukemia cells. Hairy cell leukemia can be responsible for polyarthritis due to immunity-drive inflammation, which can precede or follow the clinical onset of leukemic symptoms and usually presents as RA [7]. *PCDH9 *has not been reported as a RA susceptibility locus, suggesting it is a new candidate gene.

The next most strongly associated SNP is SNP_A-1732768, located at 142.8 Mb on chromosome 2. This SNP is ~15 Mb away from the linkage region identified through linkage analysis in Caucasian families in the North American Rheumatoid Arthritis Consortium [8]. In addition, rs4834009 (chromosome 4, 126.3 Mb), rs10520893 (chromosome 5, 23.7 Mb), and rs10509272 (chromosome 10, 67.8 Mb), are all within ~15 Mb of the linkage regions identified by Amos et al. [8]. Although not reaching genome-wide significance, several other SNPs showed trend of association, including SNPs on chromosomes 6, 8, 11, 12, 16, 17, and 20.

Unexpectedly, we did not observe significant association between RA and *PTPN22*, despite that the association with *PTPN22 *has been replicated extensively. Further examination of the data revealed that among the 42 SNPs that were examined by the HapMap, only four of them were included in the Affymetrix 100 K array set. Surprisingly, we did not observe evidence of association between RA and the HLA complex either. Among the 102 SNPs were genotyped in the HLA region, 85 passed our data quality checking, and the most strongly associated SNP had a *p*-value of 0.05. A recent study of the extended MHC region identified 6338 SNPs [9], whereas 5 only 1.6% of them are included in the Affymetrix 100 K array set. Because association analysis depends critically on the degree of LD between the tested marker and the unobserved disease locus, it is indeed not surprising that given the limited coverage of the HLA region, the current data did not support evidence of association.

## Conclusion

We conducted genome-wide linkage analysis using SNPs genotyped by the Illumina linkage panel and genome-wide association analysis using SNPs genotyped by the Affymetrix 100 K platform on a set of affected relative pairs of RA in CRAGS. Multipoint nonparametric linkage analysis identified three linkage peaks with maximum LOD score greater than 1.5. Our single marker association analysis showed strong evidence of association on chromosomes 1, 2, 4, 5, 7, 9, 10, 11, 13, 15, and 18. Several significantly associated SNPs locate at or close to the previously detected RA linkage regions, but not in the linkage peaks identified in the CRAGS.

For the well-known RA-susceptibility loci-*HLA-DRB1 *and *PTPN22*-we did not find evidence of association. Further examination of the data revealed that both regions are not well covered by the Affymetrix 100 K platform. Another possible reason is that the sample size available to this investigation is limited. Although genome-wide association is a promising approach to search susceptibility genes for complex diseases, the success of this approach depends critically on several factors, including the effect size of the disease genes, LD around the disease loci, and the sample size of the study. Our results indicate that future genome-wide association studies should employ a platform that has better coverage across the genome.

## Competing interests

The author(s) declare that they have no competing interests.
